# Reannotation-Free Automatic Recognition Methods for Empty Camera Trap Images Based on MegaDetector Optimization

**DOI:** 10.3390/ani16162609

**Published:** 2026-08-20

**Authors:** Mei Zhang, Jing-Xu Yao, Rong-Hai Wu, Xiao-Wei Li, Guo-Peng Ren, Wen Xiao, Deng-Qi Yang

**Affiliations:** 1 Department of Mathematics and Computer Science, Dali University, Dali 671003, China; meizi108hn@163.com (M.Z.);; 2 College of Agricultural and Biological Sciences, Dali University, Dali 671003, China; 3 Institute of Eastern-Himalaya Biodiversity Research, Dali University, Dali 671003, China; 4 Collaborative Innovation Center for the Biodiversity in the Three Parallel Rivers of China, Dali 671003, China; 5 Yunling Black-and-White Snub-Nosed Monkey Observation and Research Station of Yunnan Province, Dali 671003, China

**Keywords:** camera trap image, MegaDetector, context image similarity, weakly supervised learning, ensemble learning

## Abstract

Camera traps are widely used in wildlife surveys but often generate numerous empty images due to false triggers, which are time-consuming to filter manually. Microsoft’s MegaDetector (MD) is commonly applied for empty image filtering; however, setting its confidence threshold is challenging because a high threshold increases the false negative rate (FNR), whereas a low threshold raises the false positive rate (FPR), making it difficult to achieve a satisfactory trade-off between the two. To address this dilemma, we proposed three MD-based methods with different performance and computational requirements. For FNR-sensitive users, we proposed MD_CS, which integrated context image similarity to achieve a very low FNR while controlling the FPR. For FPR-sensitive users, we proposed MD_CL, which used MD to generate supervisory signals for training a classification model, thereby reducing the FPR while controlling the FNR. For users requiring both low FNR and low FPR, we proposed MD_CS_CL, an ensemble method that combined the strengths of MD_CS and MD_CL. Experimental results on multiple datasets showed that these three methods achieved a significantly better balance between FNR and FPR compared to the threshold adjustment method of the MD method. Specifically, MD_CS maintained an FNR of 1.8–5.9% while controlling the FPR at 4.9–8.5%; MD_CL kept the FPR at 0.4–2.6% while controlling the FNR at 3.8–8.4%; MD_CS_CL achieved an optimal balance with an FNR of 1.4–4.0% and an FPR of 0.3–0.9%. On a server equipped with an RTX 4090 GPU, the three methods processed 6.7, 5.4, and 4.0 images per second, respectively, and none required manual annotation. Consequently, the proposed methods reduce labor costs, improve work efficiency, and provide reliable technical support for large-scale wildlife monitoring by achieving a superior FNR–FPR trade-off compared to MD’s traditional threshold regulation.

## Simple Summary

Camera trapping produces massive amounts of empty images caused by misfires, which takes researchers plenty of time to sort out manually. MegaDetector is the mainstream tool to screen empty images, yet users struggle to pick an appropriate confidence threshold: a higher threshold misses more animal images, while a lower one retains too many useless empty shots, and it is hard to balance these two errors. To solve this practical problem in wildlife image processing, we designed three improved schemes built upon MegaDetector for different research demands. MD_CS targets researchers who want to avoid missing wildlife records, cutting missed detections via context similarity comparison. MD_CL fits users prioritizing fewer empty images, utilizing machine learning to lower false triggers. The combined MD_CS_CL method delivers the best overall performance for those pursuing low error rates on both sides. Tests on multiple wildlife datasets prove all three approaches outperform simple threshold tuning. They reach ideal trade-offs between missed animal images and false empty frames, run efficiently on regular GPUs, and eliminate manual labeling work. This toolkit effectively cuts screening workload and offers a practical technical solution for large-scale wildlife camera trapping surveys.

## 1. Introduction

In recent years, against the backdrop of escalating global ecosystem degradation and the pressing need for advanced monitoring techniques to address ecological challenges [1], the application of camera traps in ecological monitoring has become increasingly widespread, particularly in wildlife surveys, where it has gradually become a useful tool [2]. Camera traps offer numerous advantages, such as the absence of physical contact, non-interference with natural animal behaviors, and high cost-efficiency [3]. However, camera traps often generate a great volume of images [4,5], and the subsequent tasks of labeling, analyzing, and archiving these images require significant personnel cost and effort from ecological researchers [6]. In fact, massive empty images without animals are often captured due to false triggers when using camera traps [7,8,9]. For researchers, such a large volume of empty camera trap images is valueless to the study. Leveraging computer vision technology to identify and filter these empty images automatically can significantly reduce the effort required for manually labeling images, thereby improving the efficiency of image sorting and ecological research.

Currently, mainstream techniques for recognizing empty camera trap images can be categorized into two main approaches: classification model-based recognition methods and object detection-based recognition methods. The classification model-based approaches treat empty images as one of the categories. These methods require training deep learning models using a large set of labeled samples, which allows the model to automatically identify empty images [9,10,11,12,13,14,15]. For example, Norouzzadeh et al. [16] developed nine deep learning models for classification tasks using 1.4 million training samples to identify empty images. Their best model automatically filters out 76% of empty images in the test set, with 99.8% accuracy. Their false negative rate (FNR) is 3.8%, which means 3.8% of the animal images are misclassified as empty. Yang et al. [13] proposed an ensemble learning method to identify empty images. They used 240,000 labeling samples from the SS dataset to construct their models, which filtered out 86% of empty images automatically from the test set with 99.3% accuracy and 2.75% FNR. However, studies have shown that when using deep learning classification models to identify empty images, model performance decreases significantly on datasets that differ substantially from the training data, such as those from different ecosystems or survey contexts. This limits the adaptability of these methods [17,18,19].

Object detection technology [20,21] not only classifies images but also localizes the objects within the image. As a result, many researchers have applied object detection models to automatically identify camera trap images [22,23,24,25,26,27,28]. The object detection model outputs detection boxes with coordinates and the corresponding confidence values if the model detects objects in the input image. Generally, the higher the confidence value, the more reliable the detection results given by the model. For the empty image recognition task, object detection models are used to distinguish between empty and non-empty images based on whether an object is detected in the input image. If the model outputs a detection box with a confidence level exceeding the threshold, the input image is classified as non-empty; otherwise, it is classified as empty, which provides a potential solution for automatic recognition of empty images from different ecological contexts [29,30].

In 2019, the Microsoft AI for Earth team introduced the MegaDetector (MD) model [24], aimed at using object detection technology to assist in addressing ecological conservation and research challenges. The MD model can detect the presence of animals, humans, or vehicles in camera trap images. Due to its strong ability, this model has been used for identifying and filtering empty images in wildlife camera trap surveys [4,31]. It is capable of performing empty image recognition without retraining, even for images from different ecosystems, potentially solving the problem of identifying empty images. The MD model necessitates a confidence threshold to determine whether an object has been detected. Setting this threshold is critical. If the threshold is set too high, the FNR increases, potentially causing ecologists to miss opportunities to observe wild animals. Conversely, if the threshold is set too low, the FPR rises, which could increase the workload for ecologists in manually reviewing images [26]. Research has shown that the FPR of the MD model is negatively correlated with the confidence threshold of the detection box, whereas the FNR is often positively correlated with the confidence threshold of the detection box. For instance, Leorna and Brinkman [32] used the MD model to filter empty images in a dataset from the Arctic Alaska region. They set confidence thresholds at 0.25, 0.50, and 0.75; the corresponding FNRs were 69%, 72%, and 75%, respectively. The corresponding FPRs were 16%, 8% and 5%, respectively. Therefore, when the MD model is used directly without any auxiliary means, we are almost unable to simultaneously reduce the FPR and the FNR. This study combined the MD model, contextual similarity, and weakly supervised learning techniques to propose three reannotation-free automatic empty image recognition methods suitable for different scenarios, providing diverse solutions for users with varying computing resources and empty image filtering constraints.

## 2. Dataset and Research Methods

### 2.1. Dataset

To comprehensively evaluate the performance of the proposed method, this study utilized camera trap datasets from four distinct regions in Africa and China: Snapshot Camdeboo (SC), Snapshot Enonkishu (SE), Snapshot Kgalagadi (SKG), and Lasha Mountain (LSM) (see Table 1). Among these, the SC, SE, and SKG datasets were involved in the training of the MD model, whereas the LSM dataset was not. Each dataset included camera trap images and the corresponding annotations labeled by expert staff. These datasets contained both large animals and small animals. The four datasets included 17 to 42 species. The species composition of each dataset is shown in Table S1 of the Supporting Information. We defined images that contain animals and humans as non-empty images. All camera systems captured still images and operated continuously in a 24/7 mode, documenting animal presence across diurnal cycles and environmental conditions. SE, SC, and SKG datasets were captured by cameras with flash supplementation for night-mode capture, so the captured images are all color images, while the LSM datasets were color images during the day and gray images at night. Image datasets were acquired with spatial resolutions of 2592 × 2000 pixels (SE, SC, and SKG) and 4000 × 3000 pixels (LSM). Representative images from the four datasets are shown in Figure S1 of the Supporting Information.

### 2.2. The Reannotation-Free Automatic Recognition Framework for Empty Images

**Figure 1:**
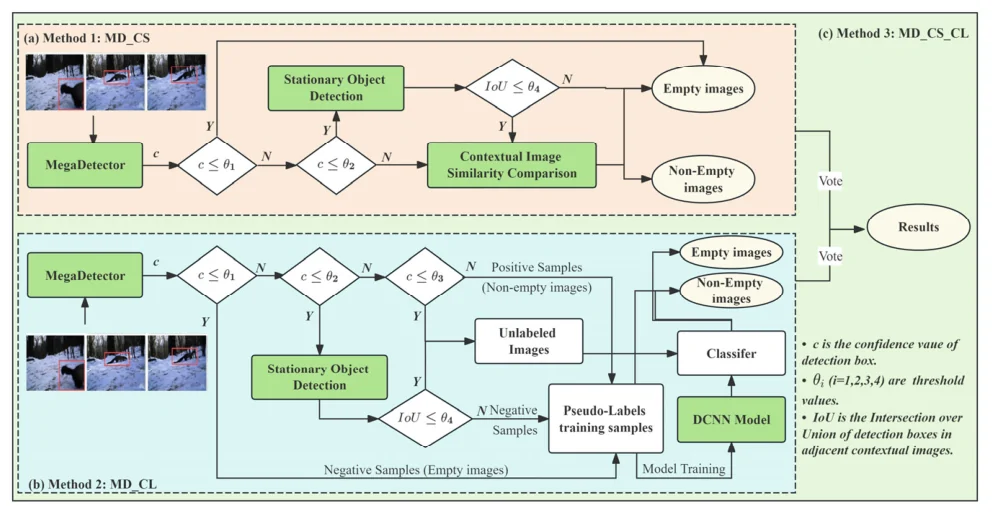
Reannotation-free automatic recognition framework for empty images.

Based on the MD model, this study introduced contextual image similarity, weakly supervised learning and ensemble learning techniques, and proposed three reannotation-free empty image recognition methods—MD_CS, MD_CL and MD_CS_CL (Figure 1)—with varying computational resource requirements and performance levels. Here, “reannotation-free” means that users are not required to manually label any new images when deploying these methods to a new survey; all training supervision is automatically generated from the MD model’s outputs. The MD_CS method was inspired by the fact that animal movement within the camera’s field of view inevitably causes pixel changes in the image, and assists in distinguishing empty and non-empty images by comparing the similarity of contextual images. The MD_CL method utilized the MD model to generate pseudo-labels, trained a classification model with these pseudo-labels, and then applied the classification model to identify images for which the MD model lacked confidence. The MD_CS_CL method integrated the MD_CS and MD_CL methods to achieve improved performance. The computational resource requirements of the three methods increased gradually, and each offered distinct performance advantages.

#### 2.2.1. Method 1: MD_CS

The MD_CS method consisted of three main modules: initial screening of the MD model, detection of stationary objects, and comparison of context image similarity (Figure 1a). First, the MD model was used to perform object detection on the input image and obtain the detection box confidence score *c.* Dual thresholds *θ*_1_ = 0.01 and *θ*_2_ = 0.20 were set to construct a hierarchical decision rule. Specifically, an empty label was assigned to the input image if *c* ≤ *θ*_1_. The input image was processed by the stationary object detection module if *θ*_1_ < *c* ≤ *θ*_2_. The input image was forwarded to the contextual image similarity module for further identification if *c* > *θ*_2_.

The stationary object detection (SOD) module checked for spatially overlapping detection boxes between the input image and subsequent frames to filter out interference from static background objects. For an input image *u*, if there existed an image *v* satisfying the following three conditions: (1) *v* was exactly the next frame of *u* in the same image sequence; (2) the confidence scores of the detection boxes in both *u* and *v* fall within the range *θ*_1_~*θ*_2_; and (3) the Intersection over Union (IoU) of these boxes was greater than θ_4_ = 0.99, then both images *u* and *v* were assigned empty-image labels. Otherwise, the input image *u* was passed to the contextual image similarity comparison module for further identification.

The contextual image similarity comparison (CS) module selected a labeled empty image *q* from the contextual images of the input image *p*. We then employed the ResNet-18 model pre-trained on ImageNet to extract the features of the corresponding regions of the highest confidence detection boxes in images p and q to calculate their cosine similarity. If the similarity was below *θ*_5_ = 0.5, or no valid empty image *q* meeting the conditions was found in the neighborhood of *p*, a non-empty label was assigned to *p*; otherwise, an empty label was assigned. Image *q* must satisfy Equation (1):

(1) $$q={min}_{\begin{array}{c} \;\;i\in N(p)\\ L_{i}=L_{p} \end{array}}|t_{i}-t_{p}|\;$$

Here, $N(p)=\{j|\exists k\in \{-1,0,1\},|t_{j}-(t_{p}+24k)|<60\}$ denoted the set of contextual images of image *p*. *t_i_*, *t_j_*, and *t_p_* represented the absolute timestamps in seconds of images *i*, *j*, and *p*, respectively. *L_i_* and *L_p_* indicated the folders (i.e., sites) where images *i* and *p* were located.

#### 2.2.2. Method 2: MD_CL

In the MD_CS method, when no valid contextual empty image *q* can be found for similarity comparison, the input image *p* is forced to receive a non-empty label, which inevitably elevates the FPR. For applications that prioritize low FPR to reduce manual verification workload, we propose a weakly supervised method, MD_CL (Figure 1b), which trains a lightweight classifier using pseudo-labels automatically derived from three sources: (i) the MD model’s detection confidence scores, (ii) the stationary object detection module, and (iii) SAM-based foreground compositing augmentation.

Unlike conventional supervised learning, MD_CL partitions each dataset into training and test subsets by confidence score rather than by random split. We first run the MD model on all images and retain the highest-confidence detection box per image. The partition rules are as follows:

- **Pseudo-empty**: images with *c* ≤ *θ*_1_ (=0.01) are directly assigned an empty label. Images with *θ*_1_ < *c* ≤ *θ*_2_ (=0.2) are examined by the SOD module; those identified as stationary (IoU > 0.99) are also labeled pseudo-empty.
- **Pseudo-animal**: images with *c* > *θ*_3_ (=0.85) are assigned a non-empty label. Each such image then undergoes a boundary integrity check: if any edge of the detection box touches the image boundary, the sample is considered incomplete and excluded from augmentation. Only images whose detection box lies fully within the frame are subjected to SAM-based foreground compositing augmentation [33], in which the extracted animal instance is randomly rotated, scaled, and composited into the lower half of a nearest-neighbor empty image to synthesize an additional training sample bearing the same pseudo-animal label. Examples of SAM-based data augmentation are provided in Supporting Information Figure S3. Non-augmented images retain their original labels.
- **Test set**: images with θ_2_ < c ≤ θ_3_ (the confidence range where MD is most uncertain), plus images with θ_1_ < c ≤ θ_2_ that SOD flags as potentially non-empty (IoU ≤ 0.99).

A ResNet-18 model [34] pre-trained on ImageNet is fine-tuned by updating all layer parameters on the pseudo-labeled training set using the Adam optimizer (initial learning rate = 0.0005, batch size = 128, loss = BCEWithLogitsLoss, 50 epochs, input resolution = 224 × 224 pixels). The best-performing checkpoint based on training accuracy was saved and used for final prediction on the test set. To mitigate variability from random initialization, training was repeated five times with independent weight initializations, and the reported metrics are the average over the five runs. The exact image counts per partition are provided in Supporting Information Table S2, and the complete training configuration is listed in Supporting Information Table S4. The final evaluation metrics (F1, FNR, FPR) are computed by comparing the predicted labels against the ground-truth labels on the complete dataset.

#### 2.2.3. Method 3: MD_CS_CL

To accommodate users who possess adequate computational resources and impose strict constraints on FNR and FPR, we developed MD_CS_CL by integrating the MD_CS and MD_CL frameworks (Figure 1c). The MD_CS_CL method assigned empty or non-empty labels through a voting strategy as in Equation (2).

(2) $$MD\_CS\_CL(x)=\{\begin{array}{c} 0,\;if\;MD\_CS(x)=MD\_CL(x)=0\\ 1,\;if\;MD\_CS(x)=MD\_CL(x)=1\\ \delta ,\;\;\;else\;\;\;\;\;\;\;\;\;\;\;\;\;\;\;\;\;\;\;\;\;\;\;\;\;\;\;\;\;\;\;\;\;\;\;\;\;\;\;\;\;\;\;\;\;\;\;\;\;\;\; \end{array}$$

Here, *x* represented the input image, “0” denoted the empty image, “1” denoted the non-empty image, and δ denoted images requiring manual identification.

### 2.3. Experimental Setup and Platform

All experiments were conducted under the Ubuntu 20.04 operating system with PyTorch 1.7.1, using two hardware configurations: an RTX 3070 Laptop GPU and a tower server with one RTX 4090 GPU (both NVIDIA Corporation, Santa Clara, CA, USA). The original MegaDetector V6 (MDV6-yolov10-e) model serves as the baseline. To comprehensively evaluate its performance across different operating points, we sweep its confidence threshold from 0.01 to 1.0 (in increments of 0.01) to generate the full FNR–FPR trade-off curve for comparison with the proposed methods.

### 2.4. Model Evaluation

To evaluate the performance of the model, this study used the false negative rate (FNR), False positive rate (FPR) [35], and empty image removal rate (EIRR), which were defined by Equations (3)–(5), respectively:

(3) $$False\;negative\;rate=\frac{FN}{TP+FN}$$

(4) $$False\;positive\;rate=\frac{FP}{TN+FP}$$

(5) $$Empty\;image\;removal\;rate=\frac{TN}{N_{e}}$$

Here, true positive (TP) referred to the number of images with non-empty ground truth labels (including images of animals, humans, or vehicles) that were correctly predicted as non-empty images. False positive (FP) indicated the number of images with empty ground truth labels that were incorrectly predicted as non-empty. True negative (TN) signified the number of images with both empty ground truth and predicted labels. False negative (FN) denoted the number of images with non-empty ground truth labels that were incorrectly predicted as empty images. *N_e_* denoted the number of empty images in the dataset.

FNR denoted the proportion of non-empty images incorrectly predicted as empty relative to the total number of non-empty images; a lower FNR suggested a reduced likelihood of missing ecological information related to the animal. Conversely, FPR directly reflected the proportion of empty images that were incorrectly predicted as non-empty; a lower FPR meant fewer empty images would require unnecessary manual review. EIRR measured the ratio of empty images removed by the proposed method to the total number of empty images in the dataset. The higher the EIRR, the greater the savings in personnel costs and effort.

## 3. Experimental Results

### 3.1. Performance

Experimental results on four distinct datasets showed that the three methods achieved promising performance across all datasets. Their EIRR values exceeded 90% on most datasets, with the sole exception of MD_CS_CL on SC (87.42%) due to its conservative voting strategy retaining a portion of images for manual review, and none of them required re-annotation of training data. Overall, each method demonstrated its own unique advantages based on the experimental outcomes (Table 2). Specifically, MD_CS achieved a relatively low FNR, e.g., 1.77% on LSM and 4.28% on SC, demonstrating strong target retention capability. However, it exhibited relatively higher FPR, e.g., 8.14% on SC and 8.45% on LSM. When both FPR and FNR were elevated—as on SKG (FPR = 5.60%, FNR = 5.92%)—MD_CS yielded the lowest F1-score among the three methods (87.54% on SKG). In contrast, MD_CL optimized feature representation via weakly supervised learning and effectively suppressed FPR, e.g., 0.40% on SKG and 0.66% on LSM, resulting in superior F1-scores (94.88% on SKG and 97.45% on LSM) and the highest EIRR of up to 99.6%. Nevertheless, MD_CL sacrificed recall, leading to a relatively high FNR, such as 8.41% on SKG. The MD_CS_CL integrated the complementary strengths of MD_CS and MD_CL, achieving the optimal balance of both low FNR (ranging from 1.41% to 4.00%) and low FPR (0.30% to 0.94%). Consequently, MD_CS_CL yielded the highest F1-score across all datasets, reaching 98.08% on SC, 97.93% on SE, 97.43% on SKG, and 98.69% on LSM, with significant reductions in both FPR and FNR compared to the individual methods. Furthermore, when using the MD_CS_CL method, if the predictions of the two base models for the same input image are inconsistent, the input image will be retained for manual review. The proportion of images requiring manual review on the four datasets was 8.76%, 7.39%, 8.14%, and 7.62%, respectively. Images flagged as δ are excluded from the TP, TN, FP, and FN counts reported in Table 2, and the reported metrics therefore reflect the performance on approximately 91–93% of images that are automatically classified. Despite its highest computational demand due to multi-model inference, MD_CS_CL delivered the most robust and reliable detection performance and provided a flexible performance-complexity gradient for practical deployment under diverse computational constraints. 95% confidence intervals for all metrics in Table 2 are reported in Table S5 of the Supporting Information.

**Figure 2:**
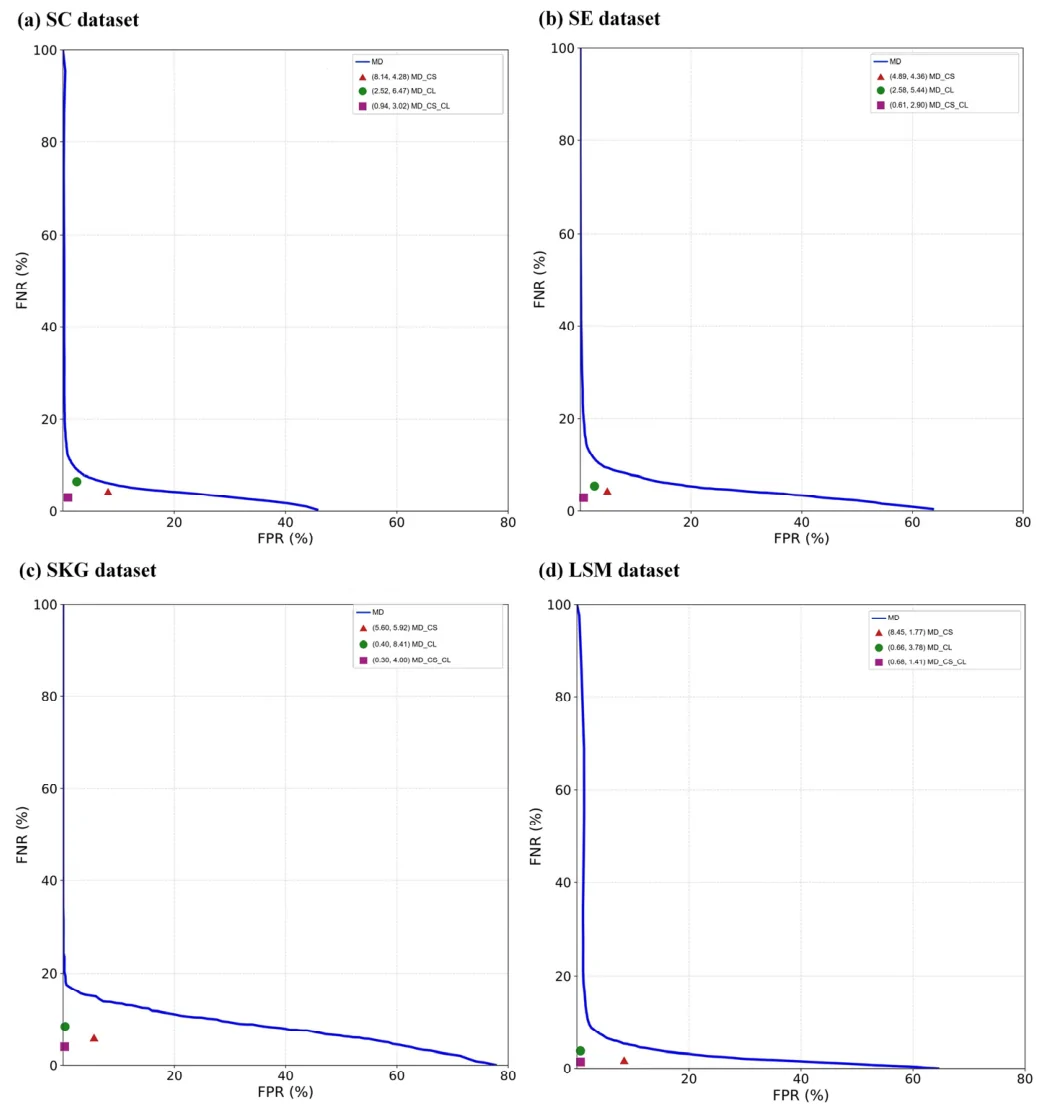
Comparison with the MD baseline model.

The FNR-FPR curves further intuitively demonstrated the overall superiority of the proposed methods over the baseline MD model (Figure 2). The MD showed a severe metric trade-off, where low FNR can only be obtained at the cost of a sharp increase in FPR. In comparison, all improved approaches achieved more reasonable results and concentrated on the optimal low-FNR and low-FPR region in the figures.

Experimental results on the three training datasets and the unseen LSM dataset showed that all three methods consistently outperform the MD baseline, suggesting that their complementary design can transfer across ecosystems. Among all schemes, the integrated MD_CS_CL method achieved the optimal overall performance in all experimental scenarios. This further verified the rationality of the contextual filtering design, pseudo-label-assisted classifier and ensemble voting mechanism in alleviating the inherent defects of the original MD model. Qualitative examples illustrating the strengths and typical failure modes of each method on the LSM dataset are provided in Figure S3 of the Supporting Information.

### 3.2. Efficiency

**Figure 3:**
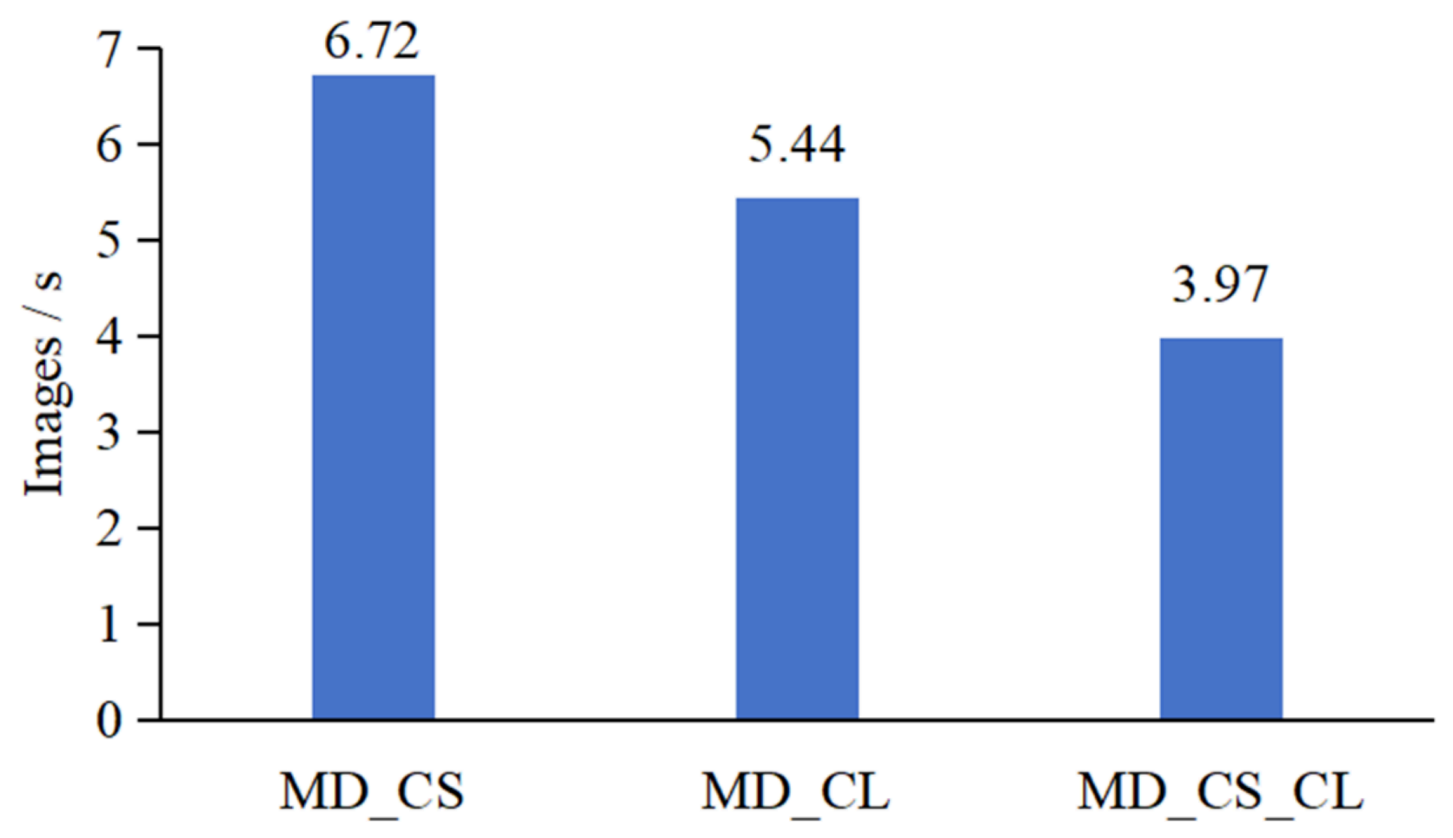
Processing efficiency of different methods on the tower server.

The three methods exhibited progressively increasing computational requirements, leading to a decreasing trend in image processing speed (Figure 3). Since MD_CS did not require model retraining, we evaluated its efficiency on two platforms: a laptop with an RTX 3070 GPU and a tower server with one RTX 4090 GPU. In contrast, MD_CL and MD_CS_CL require training the ResNet-18 model, so their efficiency was tested only on the tower server. On the laptop, MD_CS achieved an average processing speed of 3.19 images/s, which increased to 6.72 images/s on the server. On the server, MD_CL processed 5.44 images/s, while the integrated MD_CS_CL method achieved 3.97 images/s. Notably, since trained models can be reused, all speeds reported here refer to the inference stage only.

### 3.3. Ablation

Table 3 summarizes ablation tests that quantify how the SOD and CS modules jointly improve the performance of our MD_CS pipeline across four wildlife survey datasets. When only the CS module was used without SOD, the model produced extremely high FPR (FPR: 64.34–89.27%) and low empty image filtering efficiency, meaning many images containing real wildlife were incorrectly labeled as empty frames. Removing the CS module and relying solely on SOD reduced false positives moderately but still yielded poor overall classification accuracy, as static object detection alone cannot capture temporal similarities between consecutive camera trap shots to identify genuine empty images. When both modules were activated together, we observed clear performance gains across all metrics: F1 scores increased to 87.54–94.40%, FPR dropped sharply to 4.89–8.45%, and the proportion of empty images successfully filtered exceeded 90% on four datasets. While combining the two modules slightly raised FNR, this small trade-off is negligible for field surveys compared to the massive reduction in misclassified wildlife images. This outcome confirms that the two modules act synergistically: SOD filters out unchanging background clutter common in forest and grassland survey sites, and the CS module uses frame-to-frame comparisons to reliably identify true empty footage. Their combined use greatly reduces manual sorting workload for large-scale biodiversity monitoring.

Table 4 compares the performance of the MD_CL pipeline trained with and without the proposed composite data augmentation across four field survey datasets. Without augmentation, the model produced uniformly higher FNR and FPR values and lower F1 scores on all datasets. Raw training data only captures animals at natural fixed positions and sizes within frames, which restricts the model’s ability to generalize to wildlife appearing at atypical locations or scales in real camera trap footage.

Adding the foreground-compositing augmentation yielded consistent performance gains for all test sets. FNR decreased by 0.74–2.34 percentage points, while FPR showed clear declines, with the largest reduction observed on the SC dataset (from 4.01% to 2.52%). Correspondingly, F1 scores rose by 1.2–3.1%, and the empty image filtering efficiency improved across all ecological datasets. By expanding the diversity of animal sizes and frame positions presented during training, this augmentation strategy reduces misclassification errors triggered by variable animal framing in wild environments, achieving a more favorable balance between missing wildlife records and mistakenly marking wildlife images as empty. 95% confidence intervals for all metrics in Table 4 are also reported in Table S5 in the Supporting Information for details.

## 4. Discussion

### 4.1. Application and Advantages

Based on the MD model, this study constructed three re-annotation-free empty image recognition approaches, which provided customized solutions for users with diverse computational resources and performance requirements, extending the application scope of the original MD framework effectively. MD_CS performed fast inference with low FNR by introducing contextual image similarity comparison, making it suitable for scenarios with few computational resources, where low FNR was prioritized. MD_CL generated pseudo-supervision utilizing MD and trained a classifier to identify images with low confidence in detection boxes, which significantly reduced FPR and was appropriate for users with sufficient computing resources for model training and strict demands on FPR. MD_CS_CL integrated the advantages of both strategies and achieved simultaneous suppression of FNR and FPR, delivering a superior F1-score for users with abundant computational resources. The three methods exhibited strong complementarity: MD_CS optimizes inference efficiency, MD_CL refines detection precision, and their combination achieves balanced performance, which enables flexible trade-offs between speed and accuracy in practical ecological monitoring applications.

Existing studies employing the MD model typically utilize a single confidence threshold; for instance, some researchers set a threshold of 0.9 to filter empty camera trap images [31,32,36]. However, relying solely on a single confidence threshold makes it challenging to optimize FNR, FPR, and EIRR simultaneously. A high threshold reduces FPR but increases FNR, while a low threshold decreases FNR at the cost of a sharply elevated FPR. Without additional auxiliary strategies, it is challenging to balance FNR and FPR simultaneously. Some existing classification-based methods require extensive manual annotation for training [9,11,13,14,16]. Although such supervised methods can achieve low FPR, they rely on large-scale manual labeling and considerable computing resources to train models. Another lightweight method [37], the Sherlock algorithm, performed well on a small-scale dataset containing only badgers and empty images (15,073 images), with an FNR of 11.3%, FPR of 7.9%, and EIRR of 92.13%. However, this method suffers from severe performance degradation and extremely high FPR when applied to multi-species scenarios, showing poor generalization. More recently, Mononen et al. [38] proposed an active ensemble classifier that uses movement-masked images from sequences for empty-image filtering, achieving cross-habitat generalization across eight global sites. However, their method relies on active learning and requires users to manually annotate selected samples for each new camera deployment. In contrast, the three methods proposed in this work achieved favorable trade-offs among FNR, FPR, and EIRR without additional manual re-annotation and exhibited more stable performance and stronger generalization across diverse multi-species datasets.

### 4.2. Threshold Sensitivity

A series of thresholds were empirically determined in this work, including confidence thresholds *θ*_1_ = 0.01, *θ*_2_ = 0.2, *θ*_3_ = 0.85, an IoU threshold *θ*_4_ = 0.99, and a similarity threshold *θ*_5_ = 0.5. Systematic sensitivity analyses were performed to select appropriate values for these thresholds (See Table S6 and Table S7 in Supporting Information for details). For *θ*_1_, experimental comparisons showed that increasing the threshold leads to a higher FNR, while an excessively low value generates an overwhelming number of candidate boxes, causing non-maximum suppression timeouts and unnecessary computational overhead. Thus, *θ*_1_ = 0.01 was chosen to achieve a balance between FNR and efficiency. For *θ*_2_, we adopted the default parameter value of 0.2 predefined in the MD source code, which served as the fallback setting when no custom parameter was specified and was an empirical value summarized by the MD development team via extensive tests. The experimental results further indicated that our method achieved satisfactory performance across all four datasets when setting *θ*_2_ = 0.20. For *θ*_3_, tests within the range of 0.8 to 0.9 demonstrated that *θ*_3_ = 0.85 provides the best trade-off between FPR control and overall performance stability. For the IoU threshold *θ*_4_, a strict value of 0.99 was employed to identify stationary objects. This high threshold ensures that only objects with nearly unchanged bounding boxes are classified as empty images, effectively avoiding false positives caused by slight environmental fluctuations. For the similarity threshold *θ*_5_, sensitivity analysis (see Figure S4 in Supporting Information) shows that FNR decreases steadily as *θ*_5_ increases, while FPR increases gradually within the 0.4–0.6 range but rises sharply once *θ*_5_ exceeds 0.6. Therefore, 0.5 was selected as the optimal threshold, balancing FNR reduction against FPR control. All thresholds were determined using the SC, SE, and SKG datasets and applied without modification to the unseen LSM dataset, which nonetheless achieved strong performance (e.g., MD_CS: F1 = 91.78%, FPR = 8.45%), confirming that the selected values transfer reasonably across domains.

### 4.3. Selection of Detection Boxes

When using the MD model, we perform a bounding box filtering operation on each image. We only retained the bounding box with the highest confidence for each image. However, there is a scenario where the high-confidence bounding box is a false positive, and the ignored low-confidence bounding box actually contains animals. When this occurs, and the *c* is greater than *θ*_1_, the results may not be correct, potentially leading to false negatives. Fortunately, the likelihood of such cases is low: we randomly selected 1000 images from the SKG dataset that contained multiple detection boxes with a confidence level exceeding *θ*_1_. The statistics indicated that the probability of such an event occurring is merely 0.2%. Therefore, when the MD model generates multiple detection boxes for the input image, the impact of selecting the detection box with the highest confidence level on the final recognition result is very limited.

### 4.4. Limitation and Future Work

Despite the favorable performance and practical value of the proposed methods, several limitations remain. MD_CS relies on temporal and scene consistency, so its performance may degrade under frequent environmental variations such as lighting shifts, vegetation sway, or harsh weather, leading to higher FNR/FPR. It also lacks strong semantic discrimination for ambiguous scenes. Moreover, MD_CL partitions data by confidence score rather than by camera site or temporal sequence, which means images from the same camera deployment, and potentially from the same trigger event, may appear in both training and test sets. MD_CL is also limited by the quality of MD-generated pseudo-labels, which may propagate noise and errors into the classifier, particularly for low-quality or occluded images. A validation against expert annotations across all four datasets showed that 99.3% of pseudo-labels are correct (Supporting Information Table S3). We note, however, that pseudo-labels assigned by SAM-augmented samples were not included in this validation; although these represent a relatively small portion of the training set, their accuracy has not been independently verified, and they may introduce some noise into training. In addition, no explicit class imbalance handling strategy (e.g., Focal Loss, class weighting, or resampling) was employed during ResNet-18 training, although the confidence-guided data partition and SAM-based augmentation partially mitigate the extreme class skew. The integrated MD_CS_CL achieves better balance but has higher computational complexity and longer inference time. Furthermore, constrained by data availability, we did not evaluate the proposed methods on more datasets unseen by the MD model during training. Although these methods achieved encouraging zero-shot transfer performance on the novel LSM dataset, threshold sensitivity may fluctuate under extreme domain shifts across habitats, camera devices and species. In addition, all three methods use the original MD model without lightweight optimization, leading to relatively low overall computational efficiency.

Future work will address these limitations to improve robustness, efficiency, and generalization. For MD_CS, we will explore established weakly supervised learning strategies to improve robustness against pseudo-label noise, including Co-teaching [39], confidence calibration [40], and label smoothing [41]. Incorporating class imbalance mitigation techniques, such as Focal Loss or class-weighted sampling, may further boost performance on datasets with extreme empty-image ratios. We also plan to examine whether partitioning by camera deployment, rather than by confidence score, improves generalization, and to evaluate the methods on additional independently collected datasets as they become available. We will also explore adaptive thresholding strategies to improve stability under domain shifts across diverse habitats and camera configurations. For the MD_CS_CL framework, we intend to develop a dynamic weighted decision mechanism to replace the conventional simple voting strategy. Finally, we will apply lightweight optimization such as pruning and quantization to the MD model to accelerate inference for real-world deployment. In parallel, we plan to release the pipeline as a publicly available software tool, enabling ecologists to deploy empty-image filtering at scale without additional annotation burden.

## 5. Conclusions

This study addresses the limitation that the standalone MD model cannot balance FNR and FPR in camera trap empty image filtering, proposing three improved MD-based methods. Evaluated on four datasets—SC, SE, SKG training sets and the unseen LSM dataset—all methods achieve promising performance without manual annotation, maintaining low FNR and FPR while automatically filtering over 90% of empty images for MD_CS and MD_CL, and over 87% for MD_CS_CL due to its conservative voting strategy. Although further evaluation across additional ecosystems would be needed to fully assess generalizability, the results on LSM—a forest environment with different camera hardware—demonstrate encouraging zero-shot transfer capability. This work significantly reduces manual sorting workload and provides a practical, efficient solution for large-scale wildlife camera trap monitoring.

Specifically, MD_CS reduces FNR through contextual image comparison; MD_CL suppresses FPR by training a classifier with automatically generated pseudo-labels to assist prediction; the integrated MD_CS_CL combines the advantages of both to reduce both FNR and FPR simultaneously. All outperform the original MD model, serving as effective complements and offering flexible options for users with diverse performance and computational demands. This work significantly alleviates the reliance on manually labeled training samples and manual removal of empty images, enabling reliable automatic filtering and providing a practical, efficient solution for long-term large-scale wildlife camera trap monitoring.

## Figures and Tables

**Table 1:** Overview of the four datasets used in this study.

	Snapshot Camdeboo	Snapshot Enonkishu	Snapshot Kgalagadi	LSM
Images total	30,227	28,544	10,222	30,609
Empty (%)	46.48	66.73	78.85	65.19
Species total	42	36	29	17
Location	South Africa	Kenya	Botswana	China
Main ecosystem	savanna	savanna	desert	forest
Number of cameras	39	21	20	15

**Table 2:** Experimental results of three methods on various datasets.

DataSet	Method	TP	TN	FP	FN	F1 (%)	FNR (%)	FPR (%)	EIRR (%)
SC	MD_CS	15,483	12,908	1143	693	94.40	4.28	8.14	91.87
	MD_CL	15,039	13,791	357	1040	95.56	6.47	2.52	97.48
	MD_CS_CL	14,720	12,284	116	459	98.08	3.02	0.94	87.42
SE	MD_CS	9082	18,116	932	414	93.10	4.36	4.89	95.11
	MD_CL	9022	18,512	491	519	94.70	5.44	2.58	97.42
	MD_CS_CL	8615	17,454	108	257	97.93	2.90	0.61	91.63
SKG	MD_CS	2034	7609	451	128	87.54	5.92	5.60	94.40
	MD_CL	1982	8026	32	182	94.88	8.41	0.40	99.60
	MD_CS_CL	1990	7295	22	83	97.43	4.00	0.30	90.51
LSM	MD_CS	10,465	18,269	1686	189	91.78	1.77	8.45	91.55
	MD_CL	10,219	19,856	132	402	97.45	3.78	0.66	99.34
	MD_CS_CL	10,050	17,959	123	144	98.69	1.41	0.68	90.00

**Table 3:** Ablation results of the SOD and CS modules for the MD_CS pipeline.

DataSets	SOD	CS	F1 (%)	FNR (%)	FPR (%)	EIRR (%)
SC	×	√	72.05	0.77	87.76	12.26
	√	×	82.20	1.06	48.13	51.88
	√	√	94.40	4.28	8.14	91.87
SE	×	√	60.55	0.56	64.34	35.66
	√	×	67.26	1.15	47.41	52.59
	√	√	93.10	4.36	4.89	95.11
SKG	×	√	42.41	2.04	70.83	29.17
	√	×	61.40	3.32	31.73	68.27
	√	√	87.54	5.92	5.60	94.40
LSM	×	√	54.39	0.20	89.27	10.73
	√	×	73.58	0.56	37.82	62.18
	√	√	91.78	1.77	8.45	91.55

**Table 4:** Performance comparison of MD_CL with and without data augmentation.

DataSet	Data Augmentation	TP	TN	FP	FN	F1 (%)	FNR (%)	FPR (%)	EIRR (%)
SC	No	14,877	13,561	567	1222	94.33	7.59	4.01	95.99
	Yes	15,039	13,791	357	1040	95.56	6.47	2.52	97.48
SE	No	8589	18,732	522	701	93.35	7.55	2.71	97.29
	Yes	9022	18,512	491	519	94.70	5.44	2.58	97.42
SKG	No	2017	7846	116	243	91.83	10.75	1.46	98.54
	Yes	1982	8026	32	182	94.88	8.41	0.40	99.60
LSM	No	10,159	19,555	414	481	95.78	4.52	2.07	97.93
	Yes	10,219	19,856	132	402	97.45	3.78	0.66	99.34

## Data Availability

The source code can be obtained on GitHub (https://anonymous.4open.science/r/Automated_Empty_Image_Detection_for_Ecological_Monitoring-8460/, accessed on 18 August 2026). Three datasets (SE, SC, SKG) used in this study are publicly available and can be accessed via https://lila.science/datasets, accessed on 18 August 2026. The Lasha Mountain (LSM) dataset was independently collected by our research team through field camera trap surveys; all image materials are original and free of copyright disputes. Access to the LSM dataset can be requested from the corresponding author upon reasonable request for academic research purposes only.

## Supplementary Materials

The following supporting information can be downloaded at: https://www.mdpi.com/article/10.3390/ani16162609/s1.
