# Supplementary material for: Reannotation-Free Automatic Recognition Methods for Empty Camera Trap Images Based on MegaDetector Optimization
Source: Animals (Basel). 2026 Aug 20;16(16):2609. doi: 10.3390/ani16162609 (PMC13509521; doi:10.3390/ani16162609)
Supplement: Supplementary file 1 [file animals-16-02609-s001.zip › animals-4473312-supplementary.pdf]

Supporting Information for

Reannotation-Free Automatic Recognition Methods for Empty Camera Trap Images Based on MegaDetector Optimization

Mei Zhang [1], Jing-Xu Yao [1], Rong-Hai Wu [1], Xiao-Wei Li [1], Guo-Peng Ren [2], Wen Xiao [3,4,5] and Deng-Qi Yang [1,3,4,*]

1 Department of Mathematics and Computer Science, Dali University, Dali 671003,

China;meizi108hn@163.com

2 College of Agricultural and Biological Sciences, Dali University, Dali 671003, China

3 Institute of Eastern-Himalaya Biodiversity Research, Dali University, Dali 671003, China

4 Collaborative Innovation Center for the Biodiversity in the Three Parallel Rivers of China, Dali 671003, China

5 Yunling Black-and-White Snub-Nosed Monkey Observation and Research Station of Yunnan Province, Dali 671003, China

* Correspondence: dengqiyang@163.com

1. Additional Information of Datasets

1.1 Additional Descriptions

1.1.1 Snapshot Camdeboo (SC)

The SC dataset was collected in the Camdeboo National Park (32°15′7″S, 24°32′0″E), which lies in South Africa's Eastern Cape Province at the intersection of four major ecological zones: Grassland, Nama Karoo, Thicket, and Savanna. The dominant ecosystem type in this park is grassland, offering a heterogeneous matrix of open plains and semi-arid vegetation. The SC dataset collected by 39 camera traps generated 30,227 images, including a total of 43 categories. Roughly 45.11% of the dataset consists of empty images.

1.1.2 Snapshot Enonkishu (SE)

This dataset was collected in the Enonkishu Conservancy (1°4′20″S, 35°16′39″E) located in Kenya, along the northern boundary of the Mara-Serengeti ecosystem. The region is characterized by a savanna ecosystem, comprising a mosaic of grassland, woodland, and human-managed landscapes that support a wide diversity of wildlife. The SE dataset, collected by 21 camera traps, generated 28,544 images, which include a total of 37 categories. Empty images—defined as those without contained animals, or humans—constitute approximately 66.73% of the dataset.

1.1.3 Snapshot Kgalagadi (SKG)

The SKG dataset was collected in the Kgalagadi Transfrontier Park (26°16′52″S, 20°36′42″E), which spans the border between South Africa and Botswana, within the southern Kalahari Desert. This area is classified as a desert ecosystem, defined by red dunes, dry riverbeds, and sparse vegetation. The SKG dataset, collected by 20 camera traps, generated 10,222 images, including a total of 30 categories, with approximately 78.53% consisting of empty images.

1.1.4 Lasha Mountain (LSM)

This dataset was collected in the Lasha Mountain region (26º20'N,99º15'E) lies in the Three Parallel Rivers region in northwestern Yunnan, China. This region

covers areas from subtropical to subalpine zones, spanning the Gaoligong Mountains, the Nu Mountains, and the Yunling Mountains. The dominant ecosystem type in this area is forest and the LSM dataset collected by 15 camera traps generated 30,609 images, including a total of 15 categories. Roughly 65.19% of the dataset consists of empty images.

When a camera trap is triggered, we refer to it as a capture event. Staff always annotated capture events and not individual images, regardless of how many photographs a capture event comprised. For example, an event contains three images. If only one of the images contains wild animals, staff would assign animal labels to all three images. We corrected this by retaining animal labels only for the images that contain animals.

1.2 Species Composition of Each Dataset

Table S1 Species Composition of Each Dataset

Species	Dataset			
	SC	SE	SKG	LSM
1	empty	empty	empty	empty
2	human	human	human	human
3	hartebeest	bird	gemsbok	Cattle
4	vervet monkey	gazelle	bird	southwest rabbits
5	black wildebeest	warthog	jackal	sheep
6	ostrich	domestic animal	fox	tree shrews
7	hare	zebra	cape hare	dog
8	black jackal	baboon	amphibians	horse
9	springbok	hippopotamus	ostrich	chipmunks
10	buffalo	spotted hyena	eland	temminck's tragopan
11	kudu	eland	brown hyena	muntjak
12	genet	waterbuck	kori bustard	paguma
13	bird	impala	steenbok	civet cats
14	eland	ostrich	honey badger	red-bellied squirrels
15	blesbok	wildebeest	duiker common	Yunnan snub-nosed monkeys
16	reedbuck	Thomson’s gazelle	bird of prey	yellow-throated martens

17	porcupine	lion	leopard	northern musk deer
18	zebra	leopard	blue Wildebeest	spoon grouse
19	gemsbok	small antelope	red hartebeest	
20	aardvark	klipspringer	kudu	
21	steenbok	bushbuck	African wild cat	
22	blue crane	duiker	red fox	
23	baboon	mongoose	suricate meerkat	
24	fox	aardvark	lion	
25	duiker common	civet	secretary bird	
26	domestic animal	porcupine	caracal	
27	meerkat	monkey	porcupine	
28	klipspringer	giraffe	cheetah	
29	tortoise	buffalo	aardvark	
30	grey rhebok	elephant	spotted hyena	
31	caracal	hartebeest		
32	bushpig	jackal		
33	kori bustard	savannah hare		
34	reptile	oribi		
35	mongoose	rhinoceros		
36	spring hare	fox		
37	cat	cheetah		
38	prey bird			
39	deer			
41	secretary bird			
42	mongooses			
43	wolf			

1.2 Representative images of four datasets

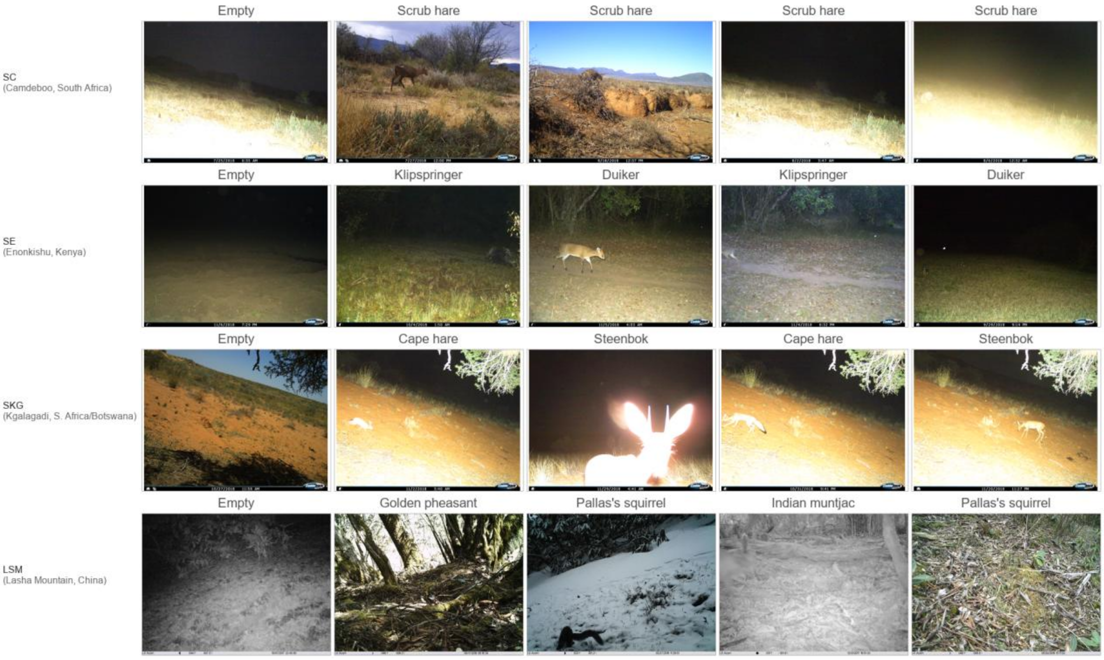

Fig. S1 Representative images from the four datasets. From top to bottom: SC (Camdeboo, South Africa, SE (Enonkishu, Kenya, SKG (Kgalagadi, South Africa/Botswana, and LSM (Lasha Mountain, China. In each row, the first column shows an empty image, and the remaining four columns show examples of small-bodied, nocturnal, or occluded challenging samples.

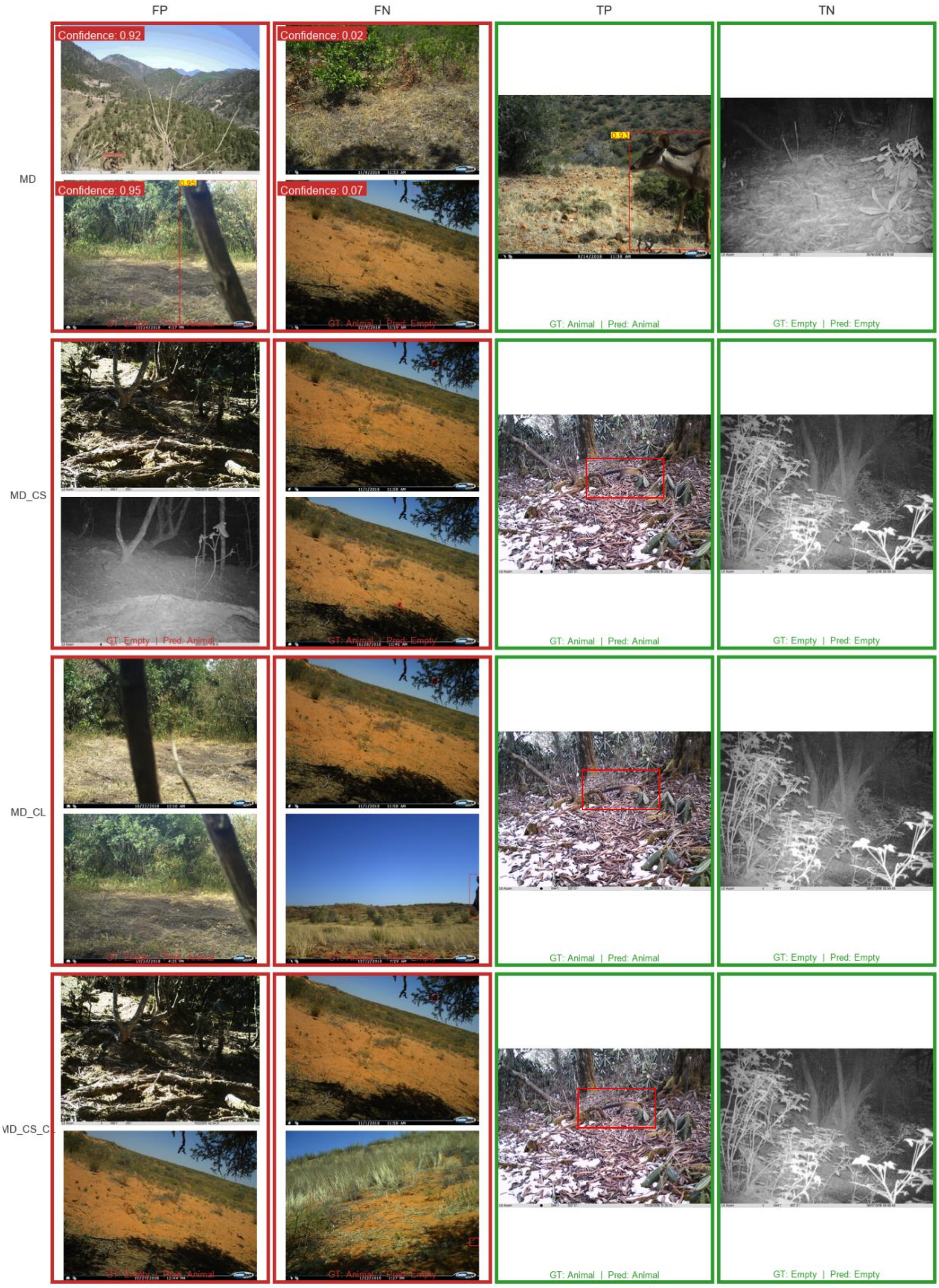

Fig. S2. TP, TN, FP, and FN examples of each method on the LSM dataset. For each image, GT (ground truth) and the method's prediction are annotated. MD results are verified against ground truth; MD_CS, MD_CL, and MD_CS_CL annotations are illustrative. V = correct, X = error, o = δ (manual review when MD_CS and MD_CL disagree).

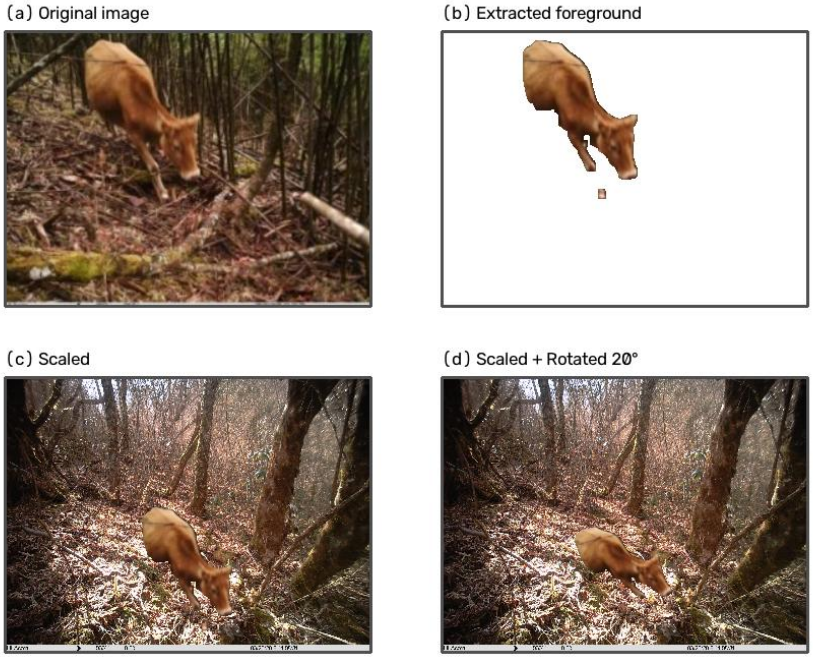

Fig.S3 Examples of SAM-based data augmentation

Table S2 Image distribution across MD_CL pseudo-labeled and testing partitions.

Dataset	Pseudo-animal	Pseudo-empty	Augmented*	Test set	Total original
SC	13,432	5,297	+4,233	11,498	30,227
SE	7,785	5,215	+3,112	15,544	28,544
SKG	1,812	3,118	+1,231	5,292	10,222
LSM	7,690	6,649	+5,322	16,270	30,609

* Synthesized via SAM-based foreground compositing augmentation.

Table S3. Validation of pseudo-labels in the training confidence regimes ($c \leqslant 0.01$, $0.01 < c \leqslant 0.2$ + SOD, $c > 0.85$) against expert annotations.

Dataset	TP	TN	FP	FN	Precision (%)	Recall (%)
SC	13,350	5,264	33	82	99.75	99.39
SE	7,742	5,158	57	43	99.27	99.45
SKG	1,757	3,111	7	55	99.60	96.96
LSM	7,656	6,594	55	34	99.29	99.56
Overall	30,505	20,127	152	214	99.50	99.30

Table S4 Training configuration for the ResNet-18 classifier in MD_CL.

**Parameter**	**Value**
Pre-trained model	ResNet-18 (ImageNet)
Fine-tuned layers	All
Input resolution	224 × 224
Optimizer	Adam
Initial learning rate	0.0005
Batch size	128
Loss function	BCEWithLogitsLoss
Total epochs	50

2. Additional results

Table S5. 95% confidence intervals for key metrics in Tables 2 and 4 (Wilson score for FPR/FNR; delta-method for F1).

Table	Dataset	Method/Aug	F1 (%)	F1 95% CI	FPR (%)	FPR 95% CI	FNR (%)	FNR 95% CI
2	SC	MD_CS	94.4	(94.15–94.65)	8.13	(7.69–8.60)	4.28	(3.98–4.61)
2	SC	MD_CL	95.56	(95.33–95.79)	2.52	(2.28–2.79)	6.47	(6.10–6.86)
2	SC	MD_CS_CL	98.08	(97.93–98.24)	0.94	(0.78–1.12)	3.02	(2.76–3.31)
2	SE	MD_CS	93.1	(92.74–93.46)	4.89	(4.60–5.21)	4.36	(3.97–4.79)
2	SE	MD_CL	94.7	(94.38–95.02)	2.58	(2.37–2.82)	5.44	(5.00–5.91)
2	SE	MD_CS_CL	97.93	(97.71–98.14)	0.61	(0.51–0.74)	2.9	(2.57–3.27)
2	SKG	MD_CS	87.54	(86.57–88.51)	5.6	(5.11–6.12)	5.92	(5.00–7.00)
2	SKG	MD_CL	94.88	(94.20–95.55)	0.4	(0.28–0.56)	8.41	(7.31–9.65)
2	SKG	MD_CS_CL	97.43	(96.94–97.92)	0.3	(0.20–0.45)	4	(3.24–4.94)
2	LSM	MD_CS	91.78	(91.41–92.14)	8.45	(8.07–8.84)	1.77	(1.54–2.04)
2	LSM	MD_CL	97.45	(97.24–97.67)	0.66	(0.56–0.78)	3.78	(3.44–4.16)
2	LSM	MD_CS_CL	98.69	(98.53–98.85)	0.68	(0.57–0.81)	1.41	(1.20–1.66)
4	SC	No	94.33	(94.07–94.58)	4.01	(3.70–4.35)	7.59	(7.19–8.01)
4	SC	Yes	95.56	(95.33–95.79)	2.52	(2.28–2.79)	6.47	(6.10–6.86)
4	SE	No	93.35	(92.99–93.71)	2.71	(2.49–2.95)	7.55	(7.03–8.10)
4	SE	Yes	94.7	(94.38–95.02)	2.58	(2.37–2.82)	5.44	(5.00–5.91)
4	SKG	No	91.83	(91.01–92.64)	1.46	(1.22–1.74)	10.75	(9.54–12.10)
4	SKG	Yes	94.88	(94.20–95.55)	0.4	(0.28–0.56)	8.41	(7.31–9.65)
4	LSM	No	95.78	(95.51–96.05)	2.07	(1.88–2.28)	4.52	(4.14–4.93)
4	LSM	Yes	97.45	(97.24–97.67)	0.66	(0.56–0.78)	3.78	(3.44–4.16)

Table S6 The results of MD_CS method with various θ_1 values

Datasets	θ_1	TP	TN	FP	FN	F1(%)	FNR(%)	FPR(%)	EIRR(%)
SC	0.01	15,483	12,908	1,143	693	94.40	4.28	8.13	91.87
	0.05	15,345	13,253	798	831	94.96	5.14	5.68	94.32
	0.1	15,340	13,266	783	838	94.98	5.18	5.57	94.43
SE	0.01	9,082	18,116	932	414	93.1	4.36	4.89	95.11
	0.05	9,068	18,354	694	428	94.17	4.51	3.64	96.36
	0.1	9,010	18,418	630	486	94.17	5.12	3.31	96.69
SKG	0.01	2,034	7,609	451	128	87.54	5.92	5.60	94.4
	0.05	2,023	7,753	307	139	90.07	6.43	3.81	96.19
	0.1	2,008	7,813	247	154	90.92	7.12	3.06	96.94
LSM	0.01	10,465	18,269	1686	189	91.78	1.77	8.45	91.55
	0.05	10,446	18,652	1303	208	93.26	1.95	6.53	93.47
	0.1	10,388	18,920	1035	266	94.11	2.5	5.19	94.81

Table S7 The results of MD_CL method with various θ_3 values（θ_1=0.01）

**DataSets**	θ_3	**TP**	**TN**	**FP**	**FN**	**F1(%)**	**FNR(%)**	**FPR(%)**	**EIRR(%)**
SC	0.8	15,004	13,326	725	1172	94.05	7.25	5.16	94.84
	0.85	14,877	13,561	567	1222	94.33	7.59	4.01	95.99
	0.9	14,588	13,661	467	1511	93.65	9.39	3.31	96.69
SE	0.8	8,602	18,682	564	696	93.18	7.49	2.93	97.07
	0.85	8,589	18,732	522	701	93.35	7.55	2.71	97.29
	0.9	8,576	18,855	402	711	93.91	7.66	2.09	97.96
SKG	0.8	2,020	7,816	163	223	91.28	9.94	2.04	98.54
	0.85	2,017	7,846	116	243	91.83	10.75	1.46	98.74
	0.9	1,950	7,850	100	322	90.24	14.17	1.26	98.74
LSM	0.8	10,219	19,341	614	435	95.12	4.08	3.08	96.92
	0.85	10,159	19,555	414	481	95.78	4.52	2.07	97.93
	0.9	9,901	19,677	388	643	95.05	6.10	**1.93**	**98.07**

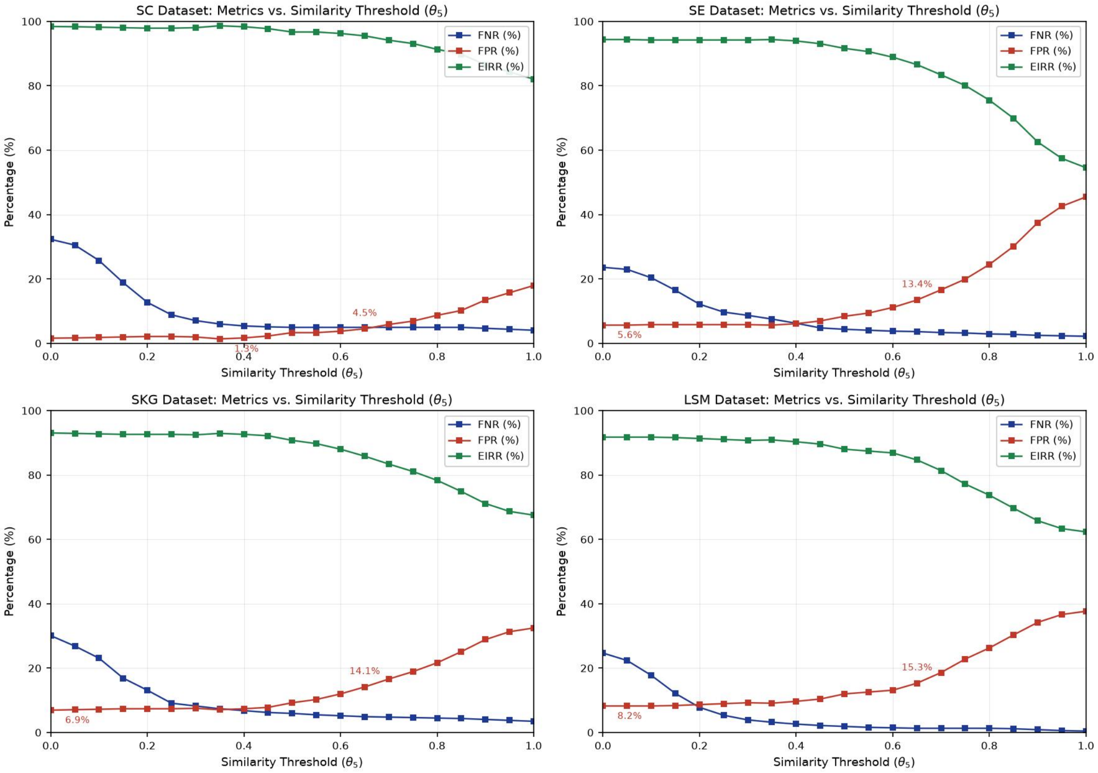

Fig.S4 The influence of similarity threshold on the performance of the MD_CS method
